# Generating meta-inferences for program improvement recommendations: mixed methods integration in joint displays for program evaluation

**DOI:** 10.3389/fpsyg.2026.1967445

**Published:** 2026-09-14

**Authors:** Laura Monkman, Dhitinut Ratnapradipa, Sergi Fàbregues, Timothy C. Guetterman

**Affiliations:** 1 Department of Family Medicine, University of Michigan, Ann Arbor, MI, United States; 2 Creighton University, School of Medicine, Omaha, NE, United States; 3 Department of Psychology, Universitat Oberta de Catalunya, Barcelona, Spain

**Keywords:** integration, joint displays, meta-inferences, mixed methods < research methodology, mixed methods analysis

## Abstract

Integration is a defining feature of mixed methods research, and the generation of meta-inferences is the overarching outcome of a mixed methods study. Despite their role in demonstrating the added value of mixed methods research, meta-inferences are frequently limited to determining fit between the results, through a comparison of quantitative and qualitative results. This article builds on a small but growing literature that has expanded conceptualization of meta-inferences by proposing an additional type that generates program recommendations. We advocate enlarging this framework to include other types of meta-inferences to give researchers additional options for addressing research questions. Thus, the aim of this paper is to demonstrate an underutilized type of meta-inference that generates program implications and recommendations. Because the goal of evaluation is the utilization of results to make program decisions, this example provides an opportunity to showcase the value of meta-inferences as applied, real world, actionable insights. We provide three examples of generating meta-inferences that focus on identifying implications and program recommendations using joint displays from an ongoing evaluation of a prevention program.

## Introduction

The defining feature of mixed methods research is integration, or the mixing of quantitative and qualitative approaches in a single study (Bazeley, 2018; Creamer, 2018). Integration occurs at multiple levels, including philosophy, research questions, the design, methods, data, and results (Guetterman and Manojlovich, 2024). Mixed methods research is well-suited to applied research, such as psychological studies in education, counseling, clinical psychology, health psychology, organizational psychology, addiction, and human behavior. The integration of qualitative and quantitative data or results, however, is particularly important in applied psychological research to generate new insights, claims, or conclusions (Creswell and Plano Clark, 2026). Conceptually, the integration of data or results produces new insight beyond what the qualitative or quantitative results alone would provide. These new insights are called meta-inferences, which reflects that their generation comes after analyzing inferences from the analysis of the qualitative and quantitative components and integrating of the two (Corrigan and Onwuegbuzie, 2020; Schoonenboom, 2022; Younas et al., 2025). Younas et al. define meta-inferences as: “overall conclusions drawn from the merging of qualitative and quantitative inferences that reveal unique insights which could not be achieved by either approach alone” (p. 2). One integration strategy to generate meta-inference is merging, whereby linked or related qualitative and quantitative results are combined to generate new inferences. The evaluation conducted for this manuscript employed merging through side-by-side joint displays, a visual technique for aligning qualitative and quantitative results on common domains to generate meta-inferences (Fetters and Guetterman, 2021; Guetterman et al., 2015). It is important to acknowledge that researchers can generate meta-inferences through other techniques such as cross-over analysis (Hitchcock and Onwuegbuzie, 2020) and qualitizing through narrative profiles (Onwuegbuzie, 2026).

The literature on meta-inferences remains underdeveloped, which may account for their poor reporting quality in published research (Younas et al., 2023). A common problem is when meta-inferences are missing or are overly simplistic comparisons of qualitative and quantitative results to determine the fit between results. Fit is an examination of whether quantitative and qualitative results are concordant (i.e., agree), discordant (i.e., disagree), or expanded (i.e., more nuanced understanding of phenomena). While determining fit may be a helpful, fit alone does not constitute a meta-inference, yet studies often report fit only without the corresponding meta-inference. Meta-inferences present an opportunity to generate practical implications and are an advantage us using a mixed methods approach.

Recent mixed methods scholarship argues that meta-inference development can take other forms. The framework developed by Younas and colleagues (2025) expanded the possibilities of meta-inferences beyond a comparison. They proposed a typology of five types of meta-inferences that are (a) relational across qualitative and quantitative data results, (b) predictive about mechanisms or events, (c) causal, and (d) elaborative of new and latent inferences as well as (e) comparative ones (Younas et al., 2025). In this manuscript, we extend their typology and propose adding a potential, yet often overlooked, type of meta-inference that seeks to develop implications and actionable recommendations to improve programs or practices in various community or organizational settings. Researchers and evaluators lack guidance on the process of developing meta-inferences beyond determining fit (i.e., whether qualitative and quantitative results agree or expand understanding). Joint displays provide a potential structured approach for integration and developing meta-inferences.

Through considering the intent of the project when conducting integrative analysis and generating meta-inferences, researchers can ensure better congruence among their study aims, methods, and results while maintaining the focus on implications for implementation. The aim of this paper is to demonstrate an underutilized type of meta-inference that generates program implications and recommendations. We illustrate how meta-inferences can be generated in ways that go beyond indicating the fit among the qualitative and quantitative results.

### Overdose prevention program background

The context for this demonstration of innovative meta-inference development is an ongoing evaluation of a local overdose prevention program funded by the Center for Disease Control and Prevention’s Overdose Data to Action (OD2A) Program in Douglas County, NE. Since 2019, OD2A has funded multi-year programs (“cooperative agreements”) in 90 jurisdictions (state and local health departments) to reduce the incidence of fatal and non-fatal drug overdoses. The core principle of OD2A is that jurisdictions should be able to craft prevention and surveillance activities responsive to communities by continually integrating local data to evolve as epidemiologically appropriate. One of the major prevention strategies of OD2A is to promote linkage to and retention in care for people who use drugs. This linkage to care strategy is intended to break down local community, public safety and healthcare siloes to implement a more coordinated system for (1) evidence-based opioid and other substance use recovery treatments, (2) training on and access to medications such as Naloxone, that reverse the effects of opioid overdose, (3) education on safer use or other forms of harm reduction, and (4) wraparound services (such as free transportation to treatment or housing assistance). In the Douglas County, NE, OD2A prevention program, partner organizations work with people who use drugs as peers and link them to various forms of care and services.

Peer navigation is when people with lived or living experience of substance use disorders support people who use drugs as peers to achieve their goals. While they often share some social characteristics with their clients and must be professionally certified in most settings, shared experience of a substance use disorder is the axis of this care delivery model (Stack et al., 2022). Moreover, according to the CDC definition, linkage to care is “a coordinated system and practice of assisting individuals with accessing care or services related to problematic opioid use” (Cruz and Chiang, 2024 p. 5).

### Evaluation framework and theory of change

The OD2A’s Linkage to Care Initiatives correspond with a theory of change whose logic model is central to our mixed methods evaluation design. The theory of change that guides OD2A Linkage to Care Initiatives focuses on outcomes at the individual and systems levels (Cruz and Chiang, 2024). To achieve intended outcomes of reduced fatal and non-fatal overdose, the program inputs include establishing access to data, establishing partnerships to support referrals, developing resources for overdose prevention, and staffing of peer navigators. A primary activity and output of the program is referral to treatment and wraparound services (i.e., whole person services). In addition, program activities and outputs relevant to this evaluation include referral partnerships and training, outreach activities to reach those at risk for overdose, overdose prevention education, and distribution of naloxone, which is a medication to reverse the effects of opioid overdose. The short-term outcomes of focus for this evaluation were increased knowledge and response to overdose, intention to enter treatment, awareness of treatment options and services, capacity of clinicians, and active referrals. The intermediate outcome is behavioral change and ultimately long-term reduced non-fatal and fatal overdose.

The model’s inputs, activities, and outputs first informed the survey constructs in our initial quantitative data collection phase. These included peer navigators’ training, active case management, referrals to different treatment and wraparound services, and client outreach. The cross-cutting actions of the CDC Evaluation Framework are collaboration, equity, and information (Kidder et al., 2024). The aims of the evaluation relevant to this manuscript are: (1) Evaluate the OD2A program process for each of the strategies and related program activities, including adherence to and quality of planned activities, to better understand implementation of initiatives (quantitative), (2) Understand the perspectives of community partner organization staff and recipients of peer support services to evaluate services provided (qualitative), and (3) Identify and explain reasons for outcome success or lack of success by integrating qualitative results of the experiences of staff and recipients (mixed methods).

## Methods

This program evaluation employs a multi-stage mixed methods evaluation design that consists of qualitative, quantitative, and mixed methods components that inform one another. Multi-stage designs combine several types of core mixed methods designs (i.e., convergent, explanatory sequential, and exploratory sequential) in the same study. The evaluation began with a needs assessment using an explanatory sequential mixed methods design to identify programmatic priorities for overdose prevention (Guetterman et al., 2026). In addition to reporting needs assessment results to program implementers and managers, these results were the basis for the ongoing evaluation, which also uses an explanatory sequential mixed methods design and is the focus of the present manuscript. This design began with surveys of peer navigators, which occurred in parallel with quantitative monthly activity tracking on OD2A-defined standard performance measures, followed by qualitative semi-structured interviews. This study has been reviewed and approved by the University of Michigan Institutional Review Board (HUM00255333).

To collaborate with different interest holders, the evaluation team formed an evaluation-community advisory board (ECAB), consisting of members of the community, community-based organization staff, and persons with lived experience with substance abuse. The ECAB provides guidance for the evaluation, such as the most relevant data to collect, recruitment and logistics of data collection, interpretation of results, and informing program recommendations. The ECAB has helped to identify priorities for the evaluation and has given feedback to the study team at different stages, such as suggesting revisions to study instruments and promoting uptake.

### Quantitative phase

For this portion of the evaluation, we collected quantitative data through multiple sources: monthly CDC-performance measure reporting and an investigator-developed survey of peer navigation staff. The CDC-mandated monthly performance measure reporting began in September 2024, which coincided with the initiation of the program. To provide time for program implementation and start-up, evaluation survey data were collected between April and June 2025 to provide sufficient time for services to be provided. Investigator-designed surveys were completed by 17 peer navigators at all three OD2A recipient partners. The survey items focused on multiple domains: 1) history in role, 2) training, 3) roles and responsibilities, 4) self-confidence and satisfaction, 5) burnout, and 6) demographics. We conducted descriptive analysis of the partner monthly reporting data and the survey data using SPSS.

### Qualitative phase

These results then informed the guide for semi-structured interviews with six peer navigators (see integration across multiple levels below) to better understand the roles, daily work, and perceptions of substance use recovery peer navigators. We invited all peer navigator staff to participate in interviews and participation was voluntary. Table 1 includes the full set of interview questions, which focused on typical workdays, what is going well, challenges, support received, training needs, and recommendations. As needed, the interviewer probed to have the participant further expand on initial or surface-level statements.

**TABLE 1:** Semi-structured interview questions for peer navigator staff

Interview question
1. To start, can you tell me a little about yourself and your work providing peer support? 2. What does a typical workday look like for you? 3. What are some things in your work as a peer navigator that you think are going well? 4. What are some things in your work as a peer navigator that are not going as well as they could? 5. What specific challenges do you encounter regularly in referring clients to services and how do you address those? These challenges can be in any area of the referral. 6. How supported do you feel in your role? 7. What training might be helpful? 8. What recommendations do you have that would improve the delivery of peer support for substance use in your organization? 9. “Blue sky” question: Assuming an ideal environment with generous resources and time, what would you change about how you do peer support work? 10. Is there anything I have not covered in this interview that you think is important for us to know?

The interview duration was 35 min to 2 h. These interviews were audio-recorded, transcribed, reviewed for accuracy, and de-identified. We analyzed transcripts using a thematic analysis approach (Braun and Clarke, 2012), consisting of reviewing the data, developing and refining a codebook, applying codes, examining textual patterns, and developing and refining themes. We used MAXQDA (Verbi GmbH, 2025) software to manage data and assist with the analysis.

Two study team members (LM and TG) used an inductive and iterative approach to codebook development. After initial coding of the same transcript the team met to discuss coding-line-by-line to develop a codebook. Codes were derived inductively directly from the data. The team identified potential themes using MAXQDA queries to examine patterns among codes. They also met to review and discuss codes and develop the evaluation themes, discussed themes with the larger research team for further development, and presented results to the ECAB.

### Integration across multiple levels

Integration occurred at the level of methods and results (Guetterman and Manojlovich, 2024). First, we used building integration through which the results of one component systematically informed the methods of data collection for the other (Fetters et al., 2013). For example, our quantitative analysis found nine total reported referrals over about 6 months for medications to treat opioid use disorder (MOUD), one of the main goals of OD2A linkage to care initiatives. This result was less than expected and subsequently informed the semi-structured interview questions with peer support providers, to explore and potentially explain the lack of referrals. Through connecting integration, we systematically used the survey results to recruit and sample staff at each of the partner organizations (Fetters et al., 2013). Third, through merging integration, we combined qualitative and quantitative results on common domains to develop meta-inferences. Specifically, we employed joint displays for analysis, a visual technique for integrating qualitative and quantitative results (Fetters and Guetterman, 2021). Primarily two study team members (LM and TG) identified programmatic implications for each key domain and developed specific recommendations to improve the program. Preliminary recommendations were then presented to the E-CAB to further refine implications and hone recommendations. The resulting side-by-side joint displays juxtapose related quantitative and qualitative results on common, logic-model aligned domains along with meta-inferences (Guetterman et al., 2015). We used these joint displays to understand program implications and develop specific, actionable recommendations for the program.

## Results

Before describing the development of three meta-inferences with implications and program recommendations, we summarize the overall results of this evaluation of the linkage to care strategy (see Supplementary Tables 1–3 for the full joint displays). The logic model also posits that outcome change should occur at the individual, organizational, and community levels (Cruz and Chiang, 2024). By integrating aggregated quantitative results with in-depth, semi-structured interviews with peer navigators, we were able to better understand the dynamic interaction of all three levels. The results showed an overall emphasis on the value of shared experience of substance use and a commitment to person-centered care with warm handoff referrals to services. Peer navigators prioritized building relationships with their clients and connecting them to the support they need, and 76% (*n* = 13) of survey participants endorsed providing emotional support to all their clients. However, the evaluation also found lower than expected referrals for harm reduction and medications for opioid use disorder (MOUD), two major OD2A linkage to care goals, as reported in meta-inference example 1 (Table 2). In addition, the results indicated a vast range of wraparound support, such as referrals for substance use treatment, physical and behavioral health needs, 12-step recovery groups, transportation, food, and housing assistance. Another key evaluation finding was that the scope of peer navigation and linkage to care for substance use varied greatly among the three community partner organizations (Table 3). At the individual-level, our evaluation found a range of peer navigator knowledge, attitudes toward and engagement with harm reduction. While tailoring the support provided is important, the results indicated that linkage to care for substance use, particularly in the areas of harm reduction and medications for recovery treatment, was dependent on the individual peer navigator and thus inconsistent. *Ad hoc* approaches to active referral at the community and systems levels then perpetuate fragmented care and unequal access to community-based overdose prevention and recovery treatments. Finally, the evaluation results indicated that navigators would like to receive further training to effectively support the needs of specific populations, as discussed in meta-inference example 3 below. For example, peer navigator education and training is a component of linkage to care that crosses domains but is especially critical for connecting clients to harm reduction services and the full range of available treatments (Table 4).

**TABLE 2:** Peer navigator linkage to care for medication for opioid use disorder (MOUD) side-by-side joint display excerpt

Quantitative results	Qualitative results	Meta-inference: implications and program recommendations
35% indicated that half of their clients are referred for MOUD; 35% indicated that few are referred for MOUD. 9 referrals for MOUD total were reported in the OD2A Local Performance Measures Tracker for the period of September 2024 to March 2025.	Linkage to Care for MOUD is implemented mainly at the individual level, sometimes misunderstood as not “treatment” or not linkage to care within the scope of their role. ● *“I’ll go down there with them to a methadone clinic and sit with them to intake process. Or if they’re contemplating methadone or Suboxone, we’ll go through the pros and cons.” (P5)* ● *“…Most of our participants will ask about treatment as opposed to a methadone clinic.” (P4)* ● *“It [MOUD] might be something that our navigators suggest if a person is having chronic relapses, abstinence is their hope and they’re overdosing…we have this tool in our back pocket.” (P09)*	● Educate Peer Support specialists to better understand MOUD, so they can counsel their clients on this option. ● Address misconceptions such as MOUD is not treatment, inferior to behavioral or other options, or a last resort, as opposed to an evidence-based approach. ● Encourage and support standardized implementation of linkage to care for the full range of opioid use disorder treatments while still centering client agency in all decision- making.

**TABLE 3:** Conflicting definitions of peer navigation and integrating referral infrastructure side-by-side joint display excerpt

Quantitative results	Qualitative results	Meta-inference: implications and program recommendations
68% indicated that they very frequently support client basic needs (food, shelter etc.) The two largest identified areas of client need were transportation (64%) and stable housing (52%).	Conflicting definitions of what peer support entails has implications for executing robust linkage to care. Differences in defining the scope, such as case management, complicate peer efforts to foster a robust referrals network. ● *“I do feel like there’s not enough coordination and enough support [between orgs].” (P5)* ● *“It’s hard to get referrals when people [from other agencies] don’t know or understand what peer support is.” (P10)* ● *“I just call it case management.” (P8)*	● Collaboration facilitates an integrated care infrastructure. ● Support collaboration between OD2A recipient peer navigator organizations, such as having regular meetings for synergies in outreach, case management, and problem-solving common issues like housing.

**TABLE 4:** Peer support training needs side-by-side joint display excerpt.

Quantitative results	Qualitative results	Meta-inference: implications and program recommendations
71% (highest rating) indicated a need for more training on working with specific populations (e.g., ethnic minorities, justice system-involved). 35% of peer support specialists identified working with people from diverse backgrounds as training area of interest. The most reported racial/ethnic group receiving Harm Reduction referrals were White (42%) followed by Hispanic/Latino (38%) in the OD2A Local Performance tracker for September 2024-March 2025.	Peer Support training would benefit from being more in-depth and tailored to different populations being served. ● *“They [peer support training provider] have a really generalized view of training and a one size fits all.”* (P5) Few peer support resources are available to serve a significant Spanish-speaking population who use drugs. ● *“A big barrier is language. There’s a large Latino population where we are.”*(P4) ● *“I find myself having to stand up and advocate for the [Latino] client base because they’re not going to understand [the translated] flyers… They’re not going to understand how you’re trying to come across.”* (P5)	● Develop special peer support modules or trainings for populations like justice-system involved, parents, unhoused, with input from all OD2A partners. ● Develop a culturally informed Peer Support training for Spanish speaking people who use drugs in partnership with the community. ● Support more Spanish-language peer support pathways or other services, including hiring peer navigators who are culturally aware and ideally from the local population’s countries of origin.

We strive for utilization-focused evaluation in which evaluation results inform program decisions (Patton, 2011). The intended outcome of the integrative analysis was to use insights as the basis for concrete steps to improve program fidelity and the overall effectiveness in linkage to care. The following three examples explain the development of meta inferences that are beyond simple comparison of qualitative and quantitative results. Rather, the meta-inferences were oriented to generating implication and recommendations for the program. In these examples, the implications and recommendations focus on prevention program improvements and are derived from mixed methods evaluation results.

### Meta-inference example 1

The joint display domains as well as the meta-inferences align with the main OD2A goals and specifically the linkage to care logic model inputs and activities. For the domain of Linkage to Care for Medication for Opioid Use Disorders (MOUD), the quantitative results indicated that while the overall number of referrals for MOUD were low, 35% of the survey sample (*n* = 6 participants) reported MOUD referrals for half of their clients, 35% for very few and the remainder, none at all. We then looked at our qualitative data to elucidate this result. The interview results showed that linkage to care for evidence-based SUD medications depends on individual peer navigator initiation at all the partner organizations. This result then anchored the meta-inference. Furthermore, such limited, peer-navigator dependent linkage to care was further explained by interview participant misperceptions. These misperceptions included one participant who explained their belief that methadone is not substance use treatment and another who said that MOUD referrals fall outside their linkage to care scope of practice. Three interviewed staff described a preference for abstinence-only recovery, yet two other participants disclosed that MOUD is part of their recovery, and both will support MOUD referral if the client expresses interest.

When asked about active referrals for MOUD, one experienced peer navigator explained: “I don’t know a ton about that. We’ve been told to… it’s unfortunate that like a doctor or clinician would have to be the one to suggest MAT [Medication Assisted Treatment] for them, not us (P10).” Based on the integrative analysis of quantitative MOUD referral results and qualitative explanations, we developed a meta-inference that peer navigators require further education on MOUD. Additional education on MOUD is needed to facilitate high-quality linkage to care that centers the client by reducing recovery intervention stigma and personal biases held by peer navigators themselves that are contrary to evidence-based care. This education gap and stigmatization of substance use recovery medication likely plays a role in the under-utilization of evidence-based treatment.

### Meta-inference example 2

Another domain concerned the practice scope of peer navigators and processes for substance use recovery active referral. One of the short-term outcomes of the OD2A logic model is developing partners to support active referral and case management. We collected quantitative data about what services clients need and what supports peer navigators provide in addition to qualitative data about how peer navigators provide support. The qualitative results expanded on the survey results that 68% of survey participants reported that they very frequently support client basic needs. In interviews, half of participants disagreed on whether case management or care coordination are within the scope of the peer navigator role. One peer navigator does not identify with the label of case manager and gave the example of a justice-involved client starting their reentry: “We will support you through that process. So I’ll sit with you while you do [paperwork] and help you do it, together. But yeah, I’m not going to do it for you…I’ll give you rides to recovery meetings” (P10). By contrast, another peer navigator who self-identified as a case manager described a larger scope of their work in supporting substance use recovery clients with needs ranging from regaining their child custody to clothing for job interviews.

Given that peer navigators are in direct service settings, conflicting definitions of the scope of the role has implications for making linkages to care, from connecting vulnerable clients to care via warm handoffs to follow-up during or afterwards. As one peer navigator observed, “I can get ‘em [clients] to the ER [emergency room], but we’re not allowed to get involved” (P8). Role ambiguity also complicates efforts to address resistance to peer-delivered support in traditional clinical settings. One peer navigator who discussed the slow rate of emergency department referrals also pointed to challenges in outreach: “Can we come in and do a presentation on peer support? Can I send you an email, give you a flyer…some of the pushback I’ve gotten with…psychiatrists…, well, ‘I went to school for 8 to 12 years to do this, and you’re just like a paid friend”’ (P10).

The meta-inference from this integrated analysis reflects the community-engaged evaluation principle of solutions originating from the peer navigators themselves: “I do feel like there’s not enough coordination and enough support [between orgs], more coordination [is needed] to give the client the best shot” (P5). Therefore, more collaboration is needed among peer navigators such as regular meetings for sharing resources and creating synergies.

### Meta-inference example 3

Peer navigator participants identified service gaps, and two participants discussed the lack of tailored, culturally responsive recovery support for several specific populations, such as Spanish-speaking clients. For instance, as reported in Table 4, the majority (71%) of peer navigators surveyed identified a need for more population-specific training, a result we corroborated with interview finding that the state-level peer navigation certification curriculum does not address tailoring services to individual needs. These integrated results in the joint display then informed our recommendation that the health department develop more focused training for peer navigation with specific demographics in Douglas County, such as unhoused or justice-involved populations.

## Discussion

The results from the program evaluation demonstrate the added value of mixed methods research in developing a more complete understanding of the prevention program, namely explaining outcomes and reasons for success or lack of success. Using mixed methods joint displays for analysis enabled the evaluation team to capture whether and how a program is working and to troubleshoot implementation issues. Understanding of the implementation and processes of the prevention program yielded implications for its effectiveness and intended outcomes.

We recommend expanding Younas et al.’s (2025) meta-inference conceptualization to include an addition type: generating implications and actionable recommendations to improve programs or practices in community or organizational settings. Through this article, we propose at least six types of meta-inferences with the addition of program recommendation meta-inferences (Table 5). We recommend this type is added to the framework of Younas et al. (2025). The joint displays from an evaluation illustrate how meta-inferences can move beyond comparison and determination of fit. Fit of integration (i.e., concordance, discordance, expansion) may be important in some situations, such as validation of one strand with another. Nevertheless, many mixed methods aims seek to generate new insights or claims. Meta-inferences should reflect the intended aim (e.g., evaluating a program and identifying improvements). In this paper, we demonstrated how meta-inferences can be focused on practical implications, such as programmatic changes, intervention improvements, or have clinical implications for workflows or standards of care, per the aims or research questions. Joint displays are a useful visual tool for developing meta-inferences. While the final column of a side-by-side joint display is often reserved for interpretation or meta-inferences, we have presented an example where the final column is explicitly generates program recommendations.

**TABLE 5:** Proposed types of meta-inferences

Type of meta-inference	Description
Relational meta-inferences	Identify the relationships and linkages across two or more inferences about cases, constructs, or variables (Younas et al., 2025)
Predictive meta-inferences	Understand tentative events, mechanisms, future observations, or patterns (Younas et al., 2025)
Causal meta-inferences	Establish cause-effect relationship based on the qualitative and quantitative inferences (Younas et al., 2025)
Comparative meta-inferences	Combines qualitative and quantitative inferences into an integrated account of the inferences (Younas et al., 2025)
Elaborative meta-inferences	Provides new and latent insights generated from the integration of qualitative and quantitative inferences, considering context (Younas et al., 2025)
Program recommendation meta-inferences	Advances implications and recommendations for program improvement (new)

Limitations of the evaluation must be acknowledged. First, we have shared a single example from a program evaluation that may not apply to other contexts. Second, regarding the specific evaluation results, definitions of peer-delivered substance use care vary widely, as does the scope of practice and related professionalization. In addition, peer navigation arguably should be tailored to the culture, population, and the individual. Variation in delivery may reflect this tailoring. Third, the data gathered for this evaluation are self-reported and may differ from data collected through observation or other methods. Nevertheless, we integrated monthly performance reporting data and surveys with interview results to develop the meta-inferences. Finally, the sample size is small yet the survey data included most peer navigation staff, and we attempted to recruit all.

Additional limitations pertain to the use of joint displays to generate meta-inferences. While joint display analysis is commonly used, other techniques such as narrative weaving, back-and-forth exchanges, following a thread, and cross-over analysis may have yielded different meta-inferences. However, these approaches are compatible with joint display analysis.

## Conclusion

The unique contribution of this manuscript is to further enlarge the conceptualization of meta-inferences beyond comparing results to determine fit of integration. We proposed meta-inferences focused on generating implications and program recommendations as an additional type beyond those proposed by Younas et al. (2025) and demonstrated the development of meta-inferences through a joint display. Future methodological scholarship should continue to innovate, expand, and refine the different types of meta-inferences. For example, meta-inferences could focus on developing clinical implications, teaching-related implications, or changes to an intervention. While labeling the type of meta-inference may have little utility in reporting, expanding the literature to acknowledge different types of meta-inferences may help researchers envision possibilities to study complex psychological research problems. Additional guidance on how to develop meta-inferences, beyond examining fit – often limited to concordance only – can enhance researchers’ ability to address their aims. Moreover, we encourage mixed methods teaching and training programs to include a broader range of possible meta-inferences. Future methodological reviews, like Younas et al.’s (2025) review are needed to showcase different types of meta-inferences to address research questions or aims.

## Data Availability

The raw data supporting the conclusions of this article will be made available by the authors, without undue reservation.
