# Supplementary material for: Generating meta-inferences for program improvement recommendations: mixed methods integration in joint displays for program evaluation
Source: Front Psychol. 2026 Sep 14;17:1967445. doi: 10.3389/fpsyg.2026.1967445 (PMC13617135; doi:10.3389/fpsyg.2026.1967445)
Supplement: Supplementary file 1 [file Supplementary_file_1.pdf]

**Supplemental Table 1. Current Linkage to Care Initiatives for Community-based Overdose Prevention and Medication for Opioid Use Disorder**

| Quantitative Results | Qualitative Results | Meta-Inferences: Implications and Program Recommendations |
|---|---|---|
| **Peer support linkage to care for harm reduction** | | |
| 41% (highest %) reported few of their clients require harm reduction, yet 35% reported most of their clients require referrals for harm reduction. | All partners distribute Narcan, but harm reduction practices and client engagement approach vary by partner.<br>• *"I have Narcan [on me] at all times… but we don't educate them on that. We tell them about it, but we don't have classes and stuff." (P8)*<br>• *"We've had…Douglas County come and provide some classes on administering Narcan, using fentanyl testing strips…then we'll always have those available." (P4)* | • Standardize harm reduction best practices across all partner organizations to ensure fidelity to community-based prevention. Example: overdose education and Narcan to clients and support persons.<br>• Ensure all partner organizations offer the full spectrum of community-based services available in Douglas County.<br>• Encourage OD2A partner organization collaboration on community-based overdose prevention. |
| **Peer support linkage to care for medication for opioid use disorder** | | |
| 35% indicated that half of their clients are referred for MOUD; 35% indicated that few are referred for MOUD.<br><br>9 referrals for MOUD were reported in the OD2A Local Performance Measures Tracker for the reporting period of September 2024 to March 2025. | Linkage to Care for MOUD is implemented mainly at the individual peer-support level and is sometimes misunderstood as not "treatment" or not part of their role.<br>• *"I'll go down there with them to a methadone clinic and sit with them to intake process. Or if they're contemplating methadone or Suboxone, we'll go through the pros and cons." (P5)*<br>• *"They basically cater to the mental health…The closest* | • Educate peer support specialists to better understand MOUD, so they can counsel their clients on this option.<br>• Address misconceptions such as MOUD is not treatment, inferior to behavioral or other options, or a last resort, as opposed to an evidence-based approach.<br>• Encourage and support standardized |

| | | |
|---|---|---|
| | *thing they've came to…is IOP…which is intensive outpatient for substance use and evals. I haven't heard of any doctor here that will write prescriptions [for MOUD].” (P8)*<br>• *“…Most of our participants will ask about treatment as opposed to a methadone clinic.” (P4)*<br>• *“I don't know a ton about that. We've been told to…it's unfortunate that like a doctor or clinician would have to be the one to suggest MAT for them, not us.” (P10)* | implementation of linkage to care for the full range of opioid use disorder treatments while still centering client agency in all decision-making. |

**Supplemental Table 2. Current Systems for Linkage to Care and Wraparound services**

<table>
<tr><th>Quantitative Results</th><th>Qualitative Results</th><th>Meta-Inferences: Implications and Program Recommendations</th></tr>
<tr><td colspan="3">The value of lived experience</td></tr>
<tr><td>75% of peer support specialists surveyed said they are often or very often “sustained by their beliefs.”</td><td>The value of shared lived experience with clients motivates and sustains peer support work.<ul><li>“I took my lived experience and I'm using it as a strength now. That really does inspire hope.” (P10)</li><li>“Our navigators are people that have experience in the communities [where] they work, so they're directly aware of the resources that are available and able to connect people to them. Oftentimes [they] have personal relationships…with providers at those organizations because they either worked there previously or…received services there.” (P9)</li></ul></td><td>By being embedded in communities, peer support specialists use their lived experience to empathize and build relationships with clients and to assist with warm handoffs.</td></tr>
<tr><td colspan="3">Post-referral processes and documentation challenges</td></tr>
<tr><td>50% indicated that they “sometimes” do post-referral processes and documentation.</td><td>Critical peer support documentation and follow-up policy is uneven across and within partner organizations.<ul><li>“We have lots of situations right now with several people where their documentation is not updated for weeks. And that's really outside of our policy.” (P9)</li><li>“Sometimes when they have a breakthrough or something happens…I want to make sure</li></ul></td><td><ul><li>Create standard operating procedures for documenting peer support in consultation with OD2A partner organizations.</li><li>Determine ways to make documentation easier.</li><li>Establish clear guidelines for OD2A partners regarding post-referral check-ins, outcome documentation, and best practices for crises like relapse.</li></ul></td></tr>
</table>

<table>
<tr><td></td><td><ul><li>when you go home that you're in the right head space.” (P5)</li><li>“I make sure as a peer support… that we have safety plans in place and relapse prevention plans, safety plans for the mental health and relapse prevention plans for the substance abuse. It's the first thing I do with every single one…[it] should be mandated, but it’s not policy.” (P5)</li></ul></td><td></td></tr>
<tr><td colspan="3">Defining the scope of peer support</td></tr>
<tr><td>68% indicated that they very frequently support client basic needs<br>(food, shelter etc.).<br><br>The two largest identified areas of client need were transportation (64%) and stable housing (52%).</td><td>Conflicting definitions of what peer support entails has implications for executing robust linkage to care. Differences in defining the scope, such as case management, complicate peer efforts to foster a robust referrals network.<ul><li>“It’s hard to get referrals when people [from other agencies] don’t know or understand what peer support is.” (P10)</li><li>“Sometimes people assume we're case managers, so we can just like do the things for the participant. And that’s not what it is either.” (P10)</li><li>“I just call it case management.” (P8)</li></ul></td><td><ul><li>Define how peer support should be implemented in terms of case management and direct services to clients.</li><li>Adopt a standard definition, guidelines for implementation of peer support by OD2A Local partners while still allowing for different missions.</li></ul></td></tr>
<tr><td></td><td>Peer support partner organizations should collaborate for an integrated infrastructure for active referrals, for synergies, and to strategize common case management issues.<ul><li>“I do feel like there's not enough coordination and enough support [between</li></ul></td><td><ul><li>Collaboration facilitates an integrated care infrastructure.</li><li>Support collaboration between OD2A recipient peer navigator organizations, such as having regular meetings for synergies in outreach, case management, and</li></ul></td></tr>
</table>

| | | |
|---|---|---|
| | *orgs], more coordination [is needed] to give the client the best shot." (P5)*<br>• *"We don't get to really do the ER. I can get 'em to the ER, but I'm not allowed to get involved… when this position first started, the ER was supposed to be calling me and telling me, Hey, we have a client." (P8)*<br>• *"Educating other agencies on what peer support is…it's hard to get referrals when people don't know or understand what peer support is." (P10)* | problem-solving common issues like housing. |

**Supplemental Table 3. Peer Support Training and Activities for Linkage to Care**

| Quantitative Results | Qualitative Results | Meta-Inferences: Implications and Program Recommendations |
|---|---|---|
| **Warm-handoffs and person-centered care** | | |
| More than half (56%) indicated that they are engaging clients to understand their needs, expectations and goals very often/every day. | Peer navigators are committed to building genuine relationships with clients and delivering person-centered linkages to care.<br>• *"Just meeting them where they're at, trying to figure out…how can we best support you… to be building that rapport.*" (P10) | Person-centered linkage to care with warm handoffs are a strength of peer support provision in Douglas County. |
| **Peer support training needs** | | |
| 71% (highest rating) indicated a need for more training on working with specific populations (e.g.,ethnic minorities, justice system-involved). | Peer Support training would benefit from being more in-depth and tailored to different populations being served.<br>• *"They [peer support training provider] have a really generalized view of training and a one size fits all."* (P5) | Develop special peer support modules or trainings for populations like justice-system involved, parents, unhoused, with input from all OD2A partners. |
| **Equity in OD2A peer support services** | | |
| 35% of peer support specialists identified working with people from diverse backgrounds as training area of interest.<br>The most reported racial/ethnic group receiving Harm Reduction referrals were White (42%) followed by Hispanic/Latino (38%) in the OD2A performance tracker for September 2024-March 2025. | Few peer support resources are available to serve a significant Spanish-speaking population who use drugs.<br>• *"A big barrier is language. There's a large Latino population where we are."* (P4)<br>• *"I find myself having to stand up and advocate for the [Latino] client base because they're not going to understand [the translated] flyers… They're not going to understand how you're trying to come across."* (P5) | • Develop a culturally informed peer support training for Spanish speaking people who use drugs in partnership with the community.<br>• Support more Spanish-language peer support pathways or other services, including hiring peer navigators who are culturally aware and ideally from the local population's countries of origin. |
